# Automating eligibility assessment and enrollment for sugammadex administration within an integrated perioperative workflow

**DOI:** 10.1093/jamiaopen/ooag021

**Published:** 2026-02-17

**Authors:** Eilon Gabel, Stephen Murray, Tristan Grogan, Theodora Wingert, Ira Hofer

**Affiliations:** Department of Anesthesiology and Perioperative Medicine, David Geffen School of Medicine at UCLA, Los Angeles, CA 90095, United States; Department of Anesthesiology and Perioperative Medicine, David Geffen School of Medicine at UCLA, Los Angeles, CA 90095, United States; Department of Medicine Statistics Core, David Geffen School of Medicine at UCLA, Los Angeles, CA 90095, United States; Department of Anesthesiology and Perioperative Medicine, David Geffen School of Medicine at UCLA, Los Angeles, CA 90095, United States; Department of Anesthesiology, Icahn School of Medicine Mount Sinai, New York City, NY 10029, United States

**Keywords:** clinical decision support, electronic health records, automated alerts, health information systems, best practice advisories, clinical trial automation, implementation science

## Abstract

**Background:**

Traditional clinical trial enrollment relies on manual screening and coordinator-led recruitment, creating scalability barriers in high-volume perioperative environments. This study evaluated whether a fully automated, electronic health record (EHR)-integrated clinical decision support (CDS) system could identify eligible patients and engage clinicians in real time without manual screening or dedicated research staff.

**Methods:**

In this prospective implementation study, predefined respiratory-risk criteria were computed within the UCLA Perioperative Data Warehouse and transmitted to the EHR via Healthcare Level Seven interfaces. Patients meeting inclusion criteria automatically triggered Best Practice Advisories (BPAs) recommending an intervention. Outcomes included system accuracy in eligibility identification, provider adherence to BPA recommendations, and technical performance metrics.

**Results:**

The automated system processed 10 592 eligible patients and achieved 51.2% provider adherence (5424 patients) to CDS prompts without coordinator involvement. BPA allocation accuracy was 69.7% among patients recovering in the post-anesthesia care unit and 59.4% when including unanticipated ICU transfers. Adherence varied significantly by care team composition, with full teams (attending + CRNA + resident) achieving 57.4% adherence compared with 42.2% for solo attendings. Workflow factors were stronger predictors of adherence than patient clinical characteristics, indicating minimal selection bias.

**Conclusions:**

Fully automated, EHR-integrated CDS can enable large-scale, workflow-embedded enrollment into implementation-focused studies. While not a substitute for research designs requiring consent or randomization, this framework demonstrates a scalable approach for automated prescreening and CDS-driven prompting that reduces reliance on coordinator-dependent processes and supports real-world implementation science.

## Background

Traditional clinical trials targeting high-risk populations often rely on manual screening, coordinator-led recruitment, and time-intensive workflows that hinder scalability. These barriers are particularly acute in perioperative environments, where time sensitivity and patient turnover challenge conventional research infrastructure. Automated systems offer the potential to streamline this process by identifying high-risk patients and supporting providers in making timely, evidence-based decisions.^1^^,^^2^ Such systems may be especially useful for workflow-integrated or pragmatic implementation studies, even though they cannot replace trial designs that require informed consent or patient-level randomization.

Creating one such system allowed us to research perioperative respiratory depression. Many studies have looked at the patient risk factors that are associated with perioperative respiratory distress; including age, sex, BMI, OSA, anemia, respiratory infections, COPD, ASA score, and prolonged surgical duration.^3–6^ Early identification and timely intervention are crucial for mitigating these risks, yet limited resources often preclude universal intensive monitoring or interventions. These operational constraints make perioperative care a natural environment in which to evaluate automated clinical decision support (CDS).

Electronic health record (EHR) systems with embedded CDS offer a solution to these challenges. Best Practice Advisories (BPAs) and similar EHR-integrated alerting mechanisms have demonstrated effectiveness in promoting evidence-based practice adoption. These systems can identify eligible research participants in real-time and prompt providers to implement interventions without disrupting clinical workflows, though the technical feasibility depends on robust data warehouse integration and seamless EHR interoperability. Despite their widespread use for guideline adherence, the role of BPAs as an automated enrollment mechanism for implementation-focused studies has been less well characterized.

Sugammadex is an alternative to neostigmine for neuromuscular blockade reversal, offering faster and more predictable recovery. Studies have shown it is associated with reduced postoperative respiratory complications and expedited discharge.^7–10^ While these clinical advantages are well-documented, the purpose of this study is not to re-evaluate sugammadex efficacy. Instead, sugammadex serves as a contextual clinical use case through which to evaluate the performance of an automated identification and prompting framework.

By leveraging an automated system to triage patients and guide treatment decisions, we explore the feasibility and scalability of conducting research studies without manual screening or dedicated research staff. This study evaluates provider adherence, workflow integration, and the operational performance of a fully automated alerting system embedded in the EHR. While sugammadex serves as the contextual intervention, the primary outcome of interest is the functionality and impact of the automation infrastructure itself. We hypothesized that a fully automated, EHR-integrated CDS system could accurately identify eligible patients and influence provider behavior at scale, while preserving clinician autonomy and without introducing systematic patient-selection bias.

## Methods

This prospective implementation study evaluated an automated clinical trial enrollment system deployed at UCLA Medical Center between February 2022 and May 2024. Data was sourced from the UCLA Department of Anesthesia and Perioperative Medicine’s Perioperative Data Warehouse (PDW), a system designed to integrate perioperative data into clinical workflows and support real-time CDS tools.^11^^,^^12^ The automated enrollment system integrated 3 core components including real-time patient risk assessment, automated BPA generation, and embedded EHR ordering workflows. This framework was designed to support workflow-integrated implementation research rather than consent-requiring randomized controlled trials.

**Figure 1.**
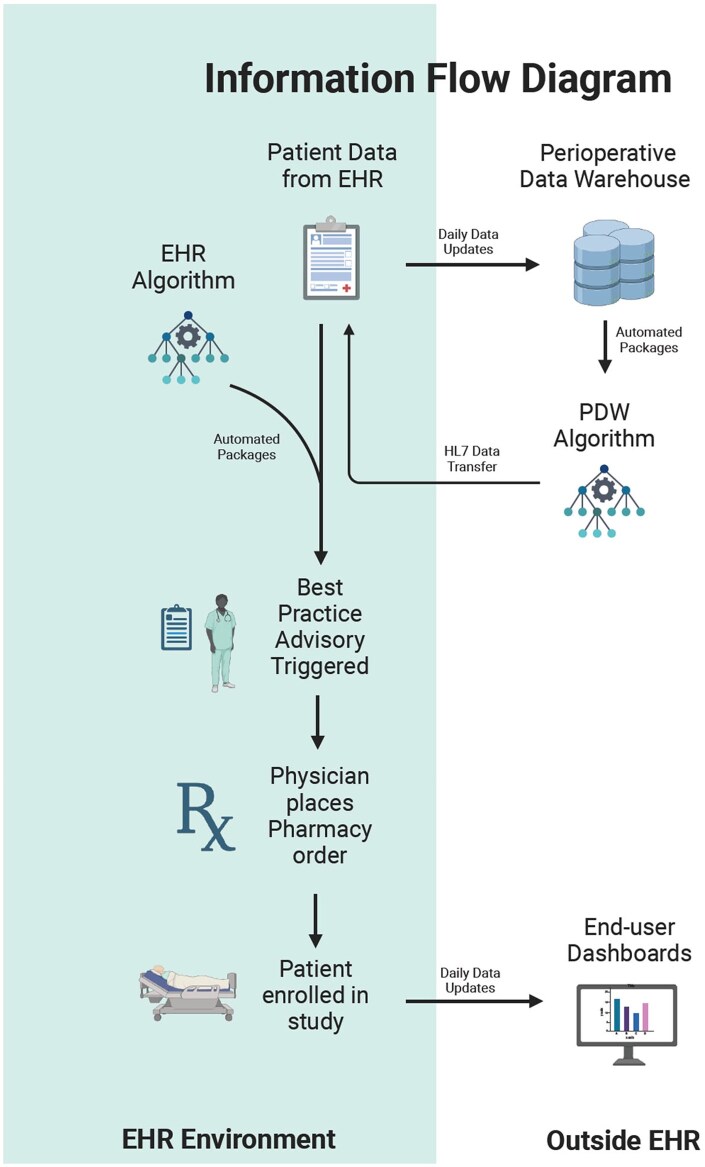
Information flow diagram. This figure illustrates the flow of information used to enable automated patient identification within the EHR. Data elements from the PDW are continuously computed and transmitted to the EHR through HL7 interfaces, where they populate structured fields used by the BPA logic. The diagram shows how PDW-derived eligibility criteria integrate with native EHR data to trigger real-time CDS alerts for clinicians. The figure reflects the operational data pathways that support automated eligibility assessment and BPA generation within routine perioperative workflows.

To detect high-risk patients, predefined inclusion criteria for perioperative respiratory risk were programmed into the PDW and transmitted to the EHR via Healthcare Level Seven (HL7) feeds. When criteria were met, a BPA was automatically triggered within the provider’s chart. This workflow is diagramed in Figure 1. All logic was executed through structured PDW fields that undergo nightly validation, cross-table consistency checks, and temporal alignment review to minimize EHR inaccuracies before alert generation. The entire process was automated with no research coordinators involved in enrollment, as all alerts and instructions were embedded directly within the EHR. Providers retained full clinical autonomy, and BPA dismissal carried no consequences.

**Figure 2.**
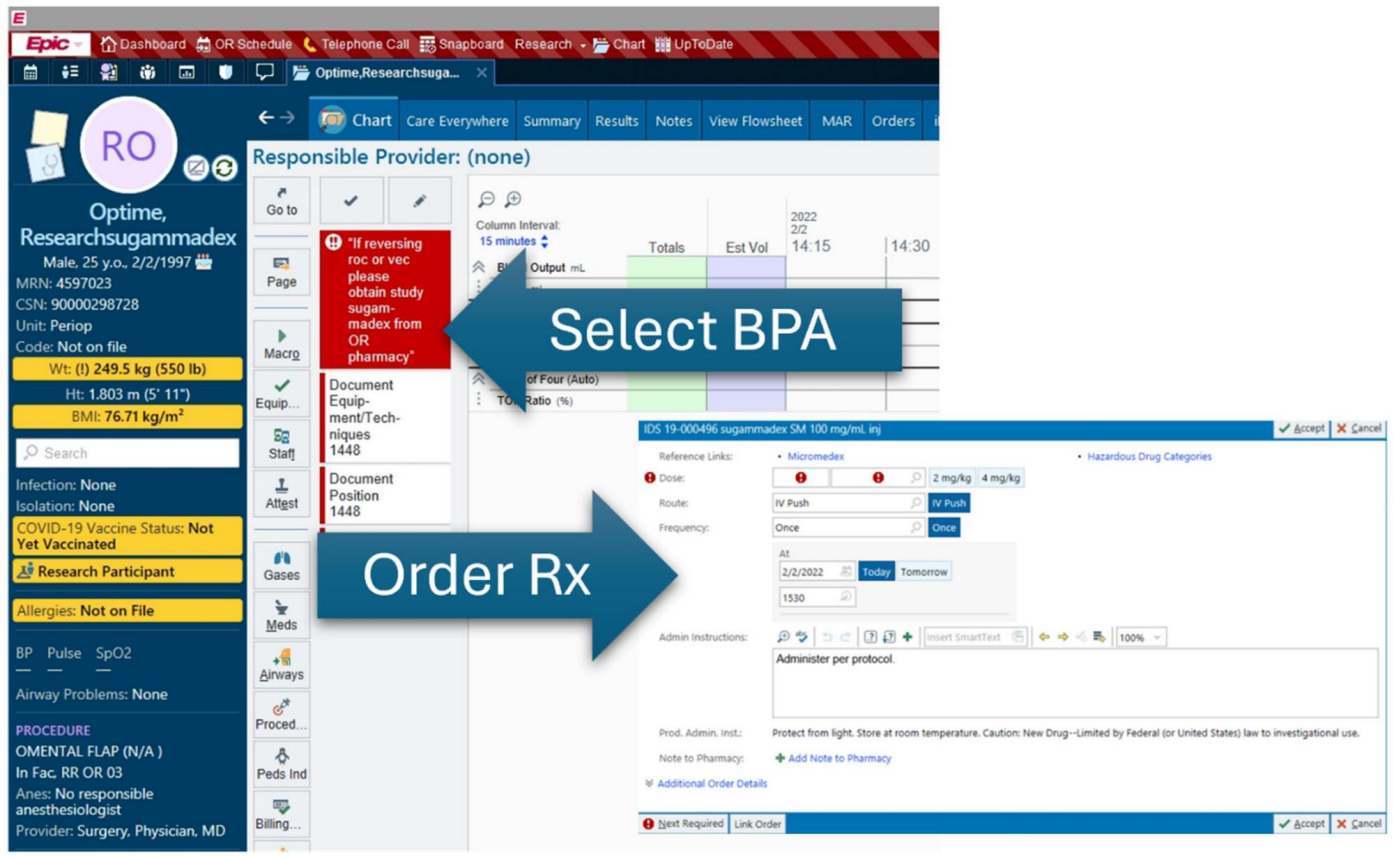
Best practice advisory interface and embedded study-drug ordering workflow. This figure displays the alert interface within the Epic EHR that appears when a patient meets the automated eligibility criteria. The alert notifies the clinician of elevated respiratory risk and provides a direct option to order the research-labeled sugammadex through an integrated workflow. The alert was designed to support unobtrusive, autonomous clinician action by embedding eligibility notification and medication ordering directly into the clinical interface, without requiring coordinator involvement. This screenshot illustrates how automated recommendations were delivered and how clinicians could initiate the study-designated order pathway within routine perioperative care.

The BPA alerts appeared in both preoperative and intraoperative navigator views, allowing providers to order study medication prior to case start or during the procedure. The BPA, shown in Figure 2, provided direct ordering capabilities for research-labeled sugammadex, which was tracked and dispensed by the OR pharmacy. Although sugammadex was also available in automated dispensing cabinets, regulatory requirements for lot tracking and manufacturer safety reporting necessitated dispensing the study-designated vials through pharmacy. System constraints limited BPA availability to main UCLA Medical Centers during operating room pharmacy hours to ensure proper medication tracking and regulatory compliance.

### Study design and regulatory approval

This study was approved by the UCLA Institutional Review Board (IRB #19-000496-AM-00002) with waiver of informed consent due to minimal risk and the observational nature of system evaluation. The trial was registered at ClinicalTrials.gov (NCT04263363) but evolved from a planned before–after comparison to a prospective implementation study due to pandemic-related delays that compromised the original design. Several elements originally included in the registry entry (eg, ventilatory management recommendations) were removed during redesign; this is now explicitly described for transparency. Provider choice remained unrestricted, with no ramifications for declining to follow BPA recommendations based on clinical judgment.

### Primary and secondary outcomes

The primary outcomes focused on system performance rather than clinical efficacy. System accuracy was defined as the proportion of BPA alerts correctly identifying eligible patients who met inclusion criteria, did not meet exclusion criteria, and recovered in the post-anesthesia care unit. Because postoperative disposition cannot be perfectly predicted preoperatively, patients admitted directly to the ICU were analyzed separately to quantify this structural limitation.

Provider adherence was measured as the proportion of appropriately allocated BPA alerts resulting in provider ordering study medication through the automated system. Secondary outcomes included technical performance metrics such as alert timing, system response rates, and workflow integration effectiveness, as well as factors associated with provider adherence patterns including care team composition and workflow variables. Additional analysis evaluated operational definitions of patient enrollment and system scalability assessment.

Patients were classified as enrolled if they triggered an appropriate BPA alert, had providers order sugammadex through the BPA-linked study pathway, and received the intervention. Patients receiving standard sugammadex outside the study workflow were classified as non-adherent to the automated system, allowing evaluation of provider response to CDS recommendations independent of clinical judgment about the intervention itself. This classification allowed isolation of behavioral response to the CDS from preference for a specific neuromuscular reversal agent.

### Patient identification algorithms

The automated patient identification system utilized inclusion criteria derived from established respiratory risk prediction models including RESPIRE, ARISCAT, and SPORC-I/II.^13–16^ The algorithm required either one major criterion or 2 minor criteria for study eligibility. Major criteria included history of obstructive or restrictive lung disease, acute respiratory infection within one month of surgery, obstructive sleep apnea, preoperative SpO_2_ less than 95%, previous history of airway pathology, and BMI greater than 40.^3^^,^^4^^,^^6^^,^^17^^,^^18^ Minor criteria encompassed multiple intubation attempts, surgery scheduled for greater than 2 hours, upper abdominal, intrathoracic, or head and neck surgery, BMI greater than 35, and preoperative hemoglobin less than 10 within 6 months.^3^^,^^19–21^ Detailed criterion definitions are provided in Table 1.

**Table 1.** Criteria for inclusion in study of sugammadex use in high-risk perioperative patients.

Criteria	Major/minor	Source	Definition	Detailed description
History of obstructive / restrictive lung disease	Major	EMR Derived	Patients who have a current or historical diagnosis of Obstructive or Restrictive Lung Disease.	Bronchitis, Emphysema, COPD, Bronchiectasis, Pulmonary diseases with fibrosis, Sarcoidosis of lung. [ICD: J40, J41-J41.8, J42, J43-J43.9, J44-J44.9, J45, J47-J47.9, J84.1, J84.9, D86]
				Preop Evaluation: COPD, Supplemental O2, Lung Transplant (historical or awaiting)
Acute respiratory infection within 1 month of surgery	Major	EMR Derived	Patients who have been diagnosed with an acute respiratory infection within 1 month prior to surgery.	Acute nasopharyngitis, Acute upper respiratory infections, Pneumonia, Bronchitis. [ICD: J00, J06-J06.9, J09, J09.x, J10-J10.89, J12-J12.9, J15-J15.9, J16-J16.8, J17, J18-J18.9, J20-J20.9]
				Preop Evaluation: Recent URI
Obstructive sleep apnea	Major	EMR Derived	Patients who have a current or historical diagnosis of Obstructive Sleep Apnea.	Obstructive Sleep Apnea. [ICD: G47.33]
				Preop Evaluation: Sleep Apnea
Preop SPO2 less than 95	Major	PDW	Patients who have an average SPO_2_ value of less than 95 in the 12 hours prior to surgery.	Average SPO_2_ < 95 in the 12 hours prior to surgery.
Previous history of airway pathology	Major	PDW	Patients who have a historical event where they had a total of more than 24 hours on a Mechanical Ventilator or a history of Tracheostomy prior to hospital admission.	More than 24 hours on a mechanical ventilator.
				Tracheostomy history prior to hospital admission, non-active.
BMI greater than 40	Major	EMR Derived	Patients who have a BMI Value greater than 40 on hospital admission.	BMI Value > 40
Multiple intubation attempts	Minor	PDW	Patients with a history of multiple endotracheal intubation events.	Patient has more than 1 Intubation attempt documented in a single case.
Surgery scheduled for greater than 2 hours	Minor	PDW	Patients with surgery scheduled for more than 2 hours.	Surgery is scheduled for more than 2 hours.
Upper abdominal/ intrathoracic/ head and neck surgery	Minor	EMR Derived	Patients who have a history of Upper Abdominal / Intrathoracic / Head and Neck Surgery.	***Required.*** Case Service is NOT “Neurosurgery” and Procedure is NOT any kind of Transplant.
				***At least 1 Required.*** Case Service is “General Surgery,” and the Procedure Name must contain one of the following: Gastric, Liver, Pancreas, Hepat, Chole.
				***At least 1 Required.*** Case Service is “Thoracic Surgery,” and the Procedure Name must contain one of the following: Pulm, Resection, Esoph, VATS, Lobe, Thoracotomy.
				***At least 1 Required.*** Case Service is “Head and Neck,” and the Procedure Name must NOT contain BOTH of the following: Mastoid, Tympano.
				***At least 1 Required.*** Laparoscopic Sleeve Gastrectomy, Laparoscopic Cholecystectomy, Laparoscopic Roux-En-Y, Removal Lap Band. [CPT code: 43775, 47562, 43644, 43774]
BMI greater than 35	Minor	EMR Derived	Patients who have a BMI Value greater than 35 on hospital admission.	BMI Value > 35
Preoperative hemoglobin less than 10 in the past 6 months	Minor	EMR Derived	Patients with a Preoperative Hemoglobin result of < 10 in the past 6 months.	Patient Lab result for “Hemoglobin, Serum” less than 10 in the last 6 months prior to surgery.

This table lists the major and minor respiratory-risk criteria used by the automated system to identify high-risk perioperative patients eligible for BPA-triggered alerts. Criteria were derived from structured EMR fields and the PDW, incorporating historical diagnoses, preoperative assessments, and surgical characteristics. Each criterion includes a detailed definition and data source to support reproducibility of the automated identification logic. These criteria were used solely to determine eligibility for automated intervention prompts and were not intended to evaluate the clinical effectiveness of sugammadex.

Exclusion criteria included patients less than 18 years old, pregnant or lactating, having pre-existing neuromuscular disease, preoperative GFR less than 30, documented sugammadex allergy, planned post-operative intubation, or direct ICU admission bypassing post-anesthesia care.^22–26^

Common predictive features across established models such as age, history of pulmonary disease, and procedure type were incorporated into our inclusion criteria, though not all variables used in published models were available in the EHR environment and required substitution with clinically appropriate surrogates. Table 2 provides comparison of features across established prediction models and criteria used in our system. Inclusion criteria were selected based on their availability in structured data fields suitable for automation, not to create a novel predictive model.

**Table 2.** Comparison of key features among the manuscript model, RESPIRE model, ARISCAT model, and SPORC models.

Model features	RESPIRE model	ARISCAT model	SPORC model	Manuscript model	Comments
**Age**	✓	✓	✓	✓	
**ASA physical status**	✓				Not typically available in time to order medication
**Functional status**	✓	✓			Not directly assessed by the EHR
**History of OSA**	✓			✓	
**Pulmonary disease history**	✓	✓	✓	✓	
**Smoking history**	✓	✓	✓		Algorithm uses any “History of Obstructive Disease” as late-stage effects from smoking
**Obesity (BMI)**	✓		✓	✓	
**Emergency surgery**	✓		✓	✓	Emergency surgery excluded
**Surgical site**	✓		✓	✓	
**Type of anesthesia**	✓		✓	✓	Non-intubated patents excluded
**Cardiovascular history**		✓	✓		No cardiovascular history accounted for by the model
**Renal function**		✓	✓	✓	GFR less than 30 excluded
**Neurological status**		✓	✓	✓	Pre-existing neuromuscular disease excluded
**Nutritional status**		✓			Anemia and obesity are the surrogate metrics of nutritional status
**Alcohol USE**		✓			Too broad to be considered for the model
**Diabetes mellitus**			✓		Too broad to be considered for the model
**Multiple intubation attempts**				✓	
**Preoperative SpO2 < 95**				✓	
**Acute respiratory infection**				✓	
**HGB < ✓ (Past 6 months)**				✓	
**Surgery > 2 hours**				✓	

This table compares the key predictor variables included in 4 perioperative respiratory-risk models—RESPIRE, ARISCAT, SPORC-I, and SPORC-II—with the eligibility criteria used in the current study.^13–16^ Although the published models were developed for risk prediction, the present study’s criteria were adapted for automated patient identification based on structured data available in the EHR and PDW. The table highlights areas of overlap, variables not reliably available for automation, and surrogate measures used when direct equivalents were unavailable. This comparison illustrates how evidence-based risk factors were operationalized for real-time CDS-triggered alerts rather than for predictive model development.

### Technical infrastructure and data processing

Inclusion data were integrated into the EHR using hybrid HL7 feeds and native chart fields, with all criteria uploaded immediately when available regardless of surgical status. If patients never underwent surgery at UCLA, the data remained hidden within the EHR, while patients requiring emergency surgery had inclusion data already available for immediate processing. The system processed patient data in real-time, triggering BPA alerts when Boolean logic evaluation confirmed eligibility criteria satisfaction. Alert generation occurred within the EHR workflow without disrupting established clinical processes. Daily validation of HL7 message integrity and PDW synchronization ensured that alert timing and content remained consistent across the study period.

Once the EHR determined that patients met inclusion criteria for CDS, an advisory was displayed to providers indicating high respiratory complication risk and study medication eligibility. Alerts were available in both preoperative chart sections for advance ordering and intraoperative navigator views for procedure-time decisions. Given the need for pharmacy staff to track research medication inventory and limited ability to disseminate study drugs to every clinical site, BPA availability was restricted to UCLA Ronald Reagan Medical Center and UCLA Santa Monica Medical Center during operating room pharmacy hours when proper tracking mechanisms were operational.

### Real-time monitoring and quality assurance

A near real-time monitoring dashboard was designed using Tableau, sourcing data directly from the PDW to track study progress, enrollment patterns, provider adherence, and adverse events requiring manufacturer reporting. The dashboard monitored BPA occurrences, medication orders, drug administration comparing study versus standard stock, and adverse event documentation. Daily review ensured immediate identification and reporting of safety events according to regulatory requirements. A representative view of this dashboard is shown in Figure S1.

Given variability in ordering and documentation practices, the definition of study enrollment required refinement during implementation. To be classified as enrolled, patients had to trigger a BPA alert, have providers order sugammadex through the BPA-linked study order rather than standard stock out of habit. Midway through the study, system-level safeguards—such as order-set constraints and automatic documentation reconciliation—were introduced to ensure accurate medication classification.

### Statistical analysis

Primary analysis employed descriptive statistics for system performance metrics with 95% CI. We compared baseline characteristics between patients who received study intervention, representing adherence to automated recommendations, versus those who did not, representing non-adherence to the CDS system. Continuous variables were reported as means with standard deviations or medians with interquartile ranges (IQRs) depending on distribution, while categorical variables were summarized as counts and percentages. Independent samples t-tests or Mann-Whitney U tests were performed for continuous variables and chi-square or Fisher’s exact tests for categorical variables as appropriate.

To evaluate whether non-adherence reflected systematic patient selection versus random provider behavior, we compared 4 key perioperative endpoints between groups including unexpected ICU transfers, reintubation rates, post-anesthesia care unit length of stay, and minimum oxygen saturation. All comparisons showed similar results, supporting the conclusion that provider adherence to BPA recommendations was not driven by underlying patient risk differences. Because adherence may be confounded by postoperative disposition, only patients recovering in the PACU and receiving rocuronium were included in multivariable modeling to limit structural bias. Multivariable logistic regression identified factors associated with provider adherence using demographic and workflow variables, with results reported as odds ratios and 95% confidence intervals. The model included only patients who received rocuronium and were not admitted directly to the ICU. Statistical significance was defined as *P* less than .05, and all analyses were performed using SAS V9.4 (SAS Institute, Cary, NC) and R V4.1 (Vienna, Austria, www.r-project.org).

## Results

The automated enrollment system successfully processed 10 592 eligible patients between February 2022 and May 2024, achieving 51.2% provider adherence (5424 patients) to automated CDS recommendations without research coordinators or enforcement mechanisms. These figures reflect real-world provider behavior, as no part of the workflow constrained or required clinicians to follow the BPA. This represents a rare, large-scale demonstration of automated clinical trial enrollment embedded directly within routine EHR workflows. Provider adherence was not actively enforced given the autonomous nature of the system, with BPAs displayed in eligible patient records prompting evidence-based intervention recommendations. Among patients receiving BPA alerts, 5424 patients were enrolled through the automated pathway while 5168 patients represented non-adherence to the CDS system, and as expected in an implementation study, the system did not track reasons for BPA dismissal.

A key overarching finding was that meaningful provider engagement with the automated CDS system could be achieved at scale without coordinator support, but that adherence was shaped primarily by workflow and team-related factors rather than patient-level clinical characteristics. Provider response patterns varied significantly by operational factors rather than patient clinical characteristics, indicating that workflow integration and team composition were primary determinants of system adoption rather than selective clinical decision-making based on patient risk profiles.

### Care team composition effects on system adoption

Provider adherence to automated recommendations varied significantly by care team structure, demonstrating the influence of collaborative workflows on CDS adoption. Cases managed by full teams including an attending physician, certified registered nurse anesthetist (CRNA), and resident achieved the highest adherence rate of 57.4% (95% CI: 54.1%-60.6%), likely reflecting collaborative decision-making processes and potential workflow handoffs. Attending-CRNA teams demonstrated similar performance with 56.9% adherence (95% CI: 55.0%-58.8%), while attending-resident teams achieved 52.6% adherence (95% CI: 51.3%-53.9%). Solo attending cases exhibited the lowest adherence at 42.2% (95% CI: 39.5%-45.0%), representing a statistically significant decrease compared to full team configurations.

**Figure 3.**
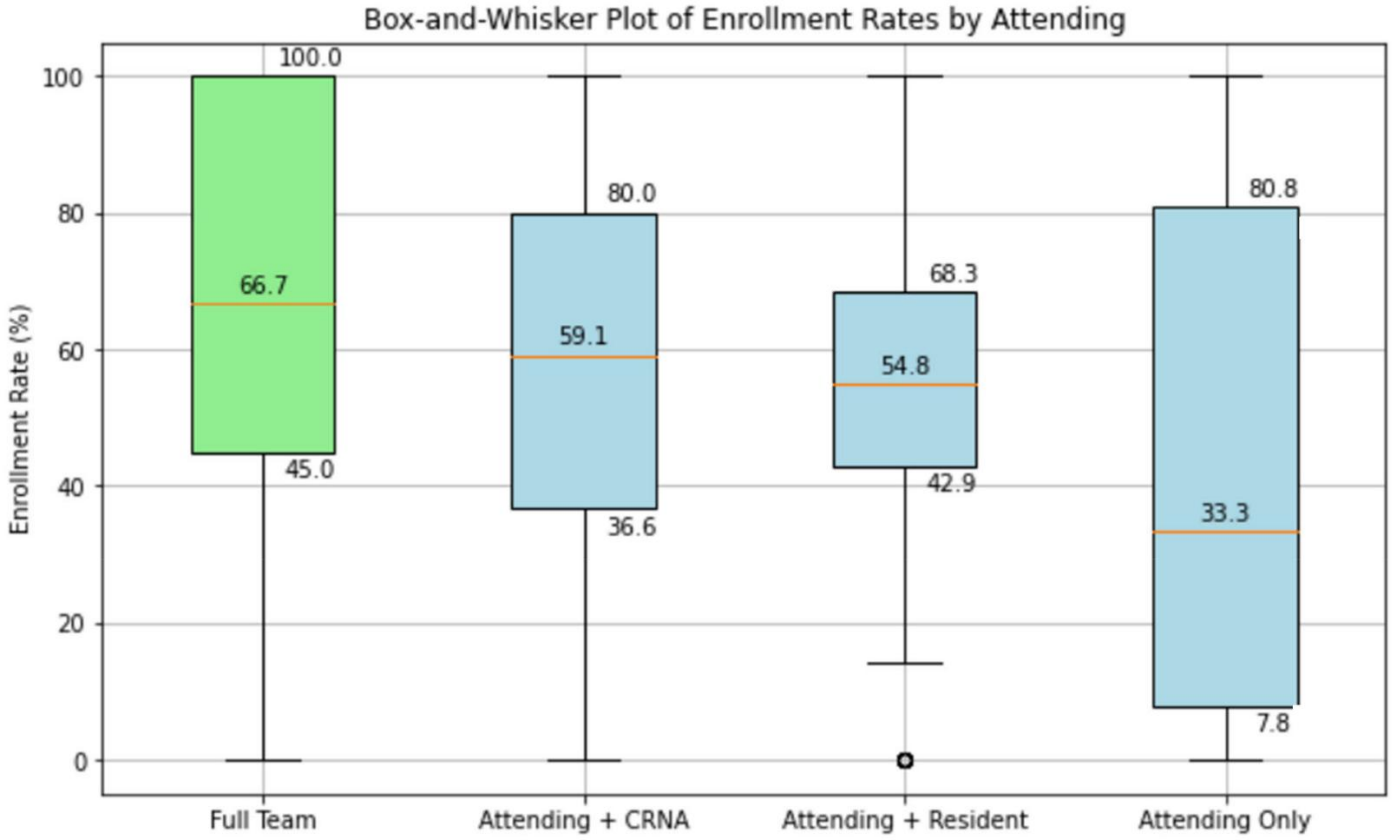
Variation in provider adherence to BPA-prompted sugammadex use across team structures. This box-and-whisker plot shows the distribution of provider adherence rates to sugammadex ordering prompted by a BPA, stratified by team composition. The 4 team structures include: “Full Team” (attending physician, CRNA, and resident physician), “Attending + CRNA,” “Attending + Resident,” and “Attending Only.” Each box represents the IQR, with the horizontal line marking the median adherence rate. Whiskers extend to 1.5 × IQR, and individual points outside the whiskers represent outliers. This visualization highlights variability in automated decision-support adoption across different staffing configurations and illustrates how collaborative team structures were associated with higher adherence.

Box-and-whisker plots shown in Figure 3 illustrate the distribution of individual attending physician adherence rates across team compositions. In full-team configurations, median provider adherence was 60.7% (IQR 50%-91%) with fewer extreme outliers observed. Solo-attending cases showed median adherence of 33.3% (IQR: 8.7%-75%) with a bimodal distribution including clusters below 30% and above 65%. Attending-resident and attending-CRNA groups displayed more centered distributions (medians 51.4% and 54.6%, respectively) but showed long tails of providers with either extremely high or exceptionally low adherence patterns. These findings confirm that while overall adherence varies by team composition, individual provider preferences and practice patterns remain powerful determinants of CDS adoption.

### Factors associated with provider adherence

Multivariable logistic regression analysis revealed that workflow factors were stronger predictors of provider adherence than patient clinical characteristics, supporting the interpretation that the automated system achieved unbiased patient identification and enrollment. Longer surgical duration increased adherence likelihood (OR = 1.35), as did procedural delays due to patient-related factors (OR = 1.36) or scheduling issues (OR = 1.29). Conversely, cases with staff-related delays were less likely to receive study intervention (OR = 0.58), indicating significant workflow influences on CDS adoption. These associations suggest that adherence reflects the realities of operational pressures, attention availability, and competing demands within the OR workflow.

Clinical factors showed modest associations with adherence patterns. Patients with hemoglobin levels less than 10 g/dL were more likely to receive intervention (OR = 2.33), as were those with moderate ASA scores (ASA 3, OR = 1.89; ASA 2, OR = 1.26) and higher BMI categories (BMI greater than 40, OR = 1.45). Certain surgical specialties including oral surgery (OR = 1.81), plastic surgery (OR = 1.53), and OB/GYN (OR = 1.31) were associated with increased adherence, while neurosurgery (OR = 0.83), thoracic surgery (OR = 0.83), and orthopedic surgery (OR = 0.69) showed lower adherence rates. Paradoxically, older patients (OR = 0.62) and those with preoperative oxygen saturation below 95% (OR = 0.48) were less likely to receive intervention despite meeting high-risk criteria, suggesting specialty-specific practice patterns rather than risk-based decision making.

### System bias assessment and patient characteristics

Comparative analysis between provider-adherent and non-adherent groups demonstrated minimal clinically significant differences in baseline characteristics, confirming that automated system deployment achieved unbiased patient identification. While some statistically significant differences emerged, such as slightly higher median age in the non-adherent group (61.0 vs. 60.0 years, *P* less than .001) and marginally lower mean BMI (29.6 vs. 30.4, *P* less than .001), these differences were not clinically meaningful. Surgical specialty distributions showed some variation with more orthopedic and otolaryngology cases in the non-adherent cohort, but overall demographic and clinical profiles remained well-balanced between groups. The full multivariable analysis is provided in Table S1.

Perioperative outcomes between provider-adherent and non-adherent groups showed no significant differences in floor-to-ICU transfers (*P* = .685), reintubation rates (*P* = 1.000), post-anesthesia care unit length of stay (*P* = .805), or minimum oxygen saturation (*P* = .172). Although mortality was higher in the provider-adherent group (13 vs. 0 events, *P* < .001), the absolute number of events was extremely low. Results of multivariable analyses are provided in Table S2 and do not suggest a causal relationship, instead reflecting the expected variability of very low-frequency outcomes. Together, these results support the conclusion that provider adherence to automated recommendations was driven by operational factors rather than selective enrollment based on patient risk assessment.

### System accuracy and technical performance

Among total BPA alerts issued, the automated system achieved 69.7% accuracy in appropriate patient identification, or 59.4% when including patients with unplanned ICU transfers that could not be predicted preoperatively. System misallocation occurred in 331 patients (4.1%) who received alerts despite meeting exclusion criteria, representing minor algorithmic errors that did not significantly impact overall study outcomes. Additionally, 1079 patients (10.3% of BPAs) were later admitted to the ICU rather than recovering in the post-anesthesia care unit as intended by the study design. Because postoperative disposition is not fully predictable from preoperative variables, this structural limitation reflects inherent challenges in preoperative CDS deployment rather than an error in the system.

Among appropriately allocated BPA recipients who recovered in the post-anesthesia care unit, 68.6% ultimately received rocuronium during their procedures, confirming that alerts effectively identified patients eligible for the targeted intervention. Despite technical limitations, most BPAs were issued appropriately, with the system correctly identifying eligible patients who would benefit from the automated CDS. Future refinements may incorporate dynamic BPA timing or real-time ICU-risk algorithms to further improve specificity, although current performance demonstrates feasibility and reliability for large-scale automated patient identification in implementation-focused research applications.

## Discussion

The most important finding of this study was successful deployment of fully automated CDS-driven enrollment for an implementation-focused study achieving 51.2% provider adherence without research coordinators. This demonstrates that clinical research infrastructure can be embedded directly into routine EHR workflows at scale. The automated system processed over 10 000 patients with 69.7% accuracy in patient identification, showing that real-time CDS can achieve meaningful provider engagement within routine workflows. Importantly, these results reflect the performance of an implementation framework rather than the testing of a therapeutic intervention, and thus should be interpreted through the lens of CDS and workflow integration rather than clinical efficacy.

Provider adherence was driven primarily by workflow factors rather than clinical characteristics, with care team composition being a stronger predictor than patient risk profiles. This pattern, combined with the minimal clinical differences between adherent and non-adherent patients, suggests that the system did not introduce systematic selection bias. This finding suggests minimal evidence of systematic selection bias while revealing important insights about CDS adoption in practice.

While clinical decision-making around neuromuscular blockade reversal is optimally informed by quantitative neuromuscular monitoring and time-to-recovery considerations. This implementation-focused study does not seek to replace physiologic assessment or define clinical indications for sugammadex versus neostigmine, but rather to evaluate the feasibility of embedding eligibility logic and enrollment workflows within routine care. In this context, the CDS framework serves as an operational and research-enabling tool that complements, rather than substitutes for, clinician judgment and quantitative monitoring when available.

### Implications for health informatics

This work demonstrates one viable strategy to address traditional barriers to clinical research scalability can be overcome through EHR integration and automated patient identification. The automated approach ensured consistent application of inclusion criteria across all eligible patients, eliminating variability inherent in manual screening processes. By embedding eligibility logic, enrollment, and intervention prompts directly into routine perioperative workflows, the system demonstrates how research-related processes can be integrated into clinical operations without additional personnel.

Key innovations include seamless integration with existing clinical workflows, real-time patient identification using established risk algorithms, automated CDS with embedded ordering capabilities, and monitoring dashboards that enable quality assurance without dedicated research staff. Rather than replacing traditional research infrastructure for trials requiring consent or randomization, this framework provides a replicable model for pragmatic CDS evaluations, implementation studies, and automated prescreening efforts. This represents a shift from parallel research workflows to embedded research capabilities within routine care.

### Clinical decision support adoption and provider behavior

Individual variation in provider adherence reveals important insights about CDS adoption. The bimodal distribution in solo attending cases, with providers showing either very high or very low adherence, suggests that effectiveness depends heavily on individual attitudes rather than system design alone. This phenomenon aligns with known patterns of alert fatigue, personalization of practice, and variable trust in EHR-generated recommendations. Care team composition effects, with full teams achieving 57.4% adherence compared to 42.2% for solo attendings, indicate that collaborative decision-making enhances CDS adoption.

The finding that high-risk patients were sometimes less likely to receive intervention despite meeting inclusion criteria suggests specialty-specific practice patterns that override algorithmic recommendations. However, the overall lack of clinical bias in system adoption supports the automated approach’s potential to achieve equitable patient identification. These results highlight an important CDS principle: system performance depends not just on algorithmic accuracy but also on the social and organizational context in which alerts are deployed.

### Limitations

Several limitations affect interpretation of these results. Automated enrollment and data collection, while minimizing human error, may have missed nuanced clinical details typically captured through manual oversight. EHR data inaccuracies, such as omissions or discrepancies, could have influenced the results. The single-institution study design and exclusion of ambulatory patients limit generalizability of these findings. Additionally, because the system depends on structured PDW and EHR fields, unstructured or free-text clinical nuance could not be incorporated into eligibility logic.

The timing of the study presented challenges. Initially designed to compare patient populations before and after sugammadex introduction, the study faced delays due to logistical complications and the COVID-19 pandemic, which extended the timeline between control and intervention cohorts. These delays may have introduced confounding variables related to temporal changes in practice patterns or patient populations. Providers bypassed the study in 48.8% of cases by using stock sugammadex, potentially introducing selection bias. However, comparison of perioperative outcomes and baseline characteristics between adherent and non-adherent patients suggests that this bypass reflected workflow realities and provider preference rather than patient selection.

The lack of clear correlation between BPA provider adherence and specific clinical features suggests that workflow factors may play a larger role than patient selection. While some clinical variables such as hemoglobin levels and higher BMI were associated with study enrollment, the influence of surgical specialty, procedural delays, and staffing-related issues indicates that provider workflow and institutional logistics likely drive decision-making more than patient-specific risk factors. This underscores the need for complementary qualitative research to understand provider motivations and alert-handling behavior.

The Epic-specific implementation may not translate directly to other EHR systems. The perioperative focus may not generalize to other clinical domains with different workflow patterns. The inability to predict unplanned ICU transfers, affecting 10.3% of alerts, represents a challenge for preoperative decision support systems that rely on predicted care pathways. The observational study design without randomization limits causal inference about system effectiveness. These limitations reflect real-world constraints inherent in embedded CDS deployment rather than shortcomings of the automation infrastructure.

### Future directions

Multi-institutional validation studies are needed to assess generalizability across diverse health systems and EHR platforms. Development of platform-agnostic interfaces would enable broader implementation. The potential for embedded randomization could enable pragmatic randomized controlled trials that operate within routine care workflows.

Implementation science research should focus on organizational and provider factors that influence CDS adoption. Mixed-methods approaches—including qualitative interviews, workflow analysis, and user-centered redesign—will be essential for identifying root causes of BPA dismissal and informing future CDS optimization. Future implementations could leverage machine learning to enhance predictive accuracy and reduce false-positive alerts, particularly for unplanned ICU transfers.

## Conclusion

This study provides evidence that fully automated CDS-driven enrollment for an implementation-focused study through EHR-integrated CDS is feasible and can achieve meaningful provider engagement at scale. By embedding eligibility logic, alert delivery, and intervention ordering directly into perioperative workflows, the system demonstrated that research-related processes can operate autonomously without coordinator involvement or manual screening. The technical framework represents a shift from resource-intensive parallel research workflows to embedded research capabilities that operate within routine care delivery. Provider adoption patterns provide important insights for optimizing CDS implementation. Specifically, CDS effectiveness depends not only on algorithmic accuracy but also on workflow context, team composition, and individual provider behavior.

The implications extend beyond clinical research to quality improvement initiatives, clinical guideline implementation, and population health management. Although this model is not a substitute for randomized controlled trials that require patient consent or investigational oversight, it provides a scalable mechanism for pragmatic CDS evaluations and automated prescreening pipelines. This work establishes automated clinical research enrollment infrastructure as a viable approach to scaling evidence-based interventions while reducing resource barriers that limit research translation into practice. Future multi-institutional studies will be essential to determine generalizability across different EHR platforms, workflow environments, and organizational cultures.

## Supplementary Material

ooag021_Supplementary_Data

## Data Availability

The data underlying this article cannot be shared publicly due to HIPAA regulations and institutional policy. The data will be shared (de-identified) on reasonable request to the corresponding author.
