# Supplementary material for: Automating eligibility assessment and enrollment for sugammadex administration within an integrated perioperative workflow
Source: JAMIA Open. 2026 Feb 17;9(1):ooag021. doi: 10.1093/jamiaopen/ooag021 (PMC12932941; doi:10.1093/jamiaopen/ooag021)
Supplement: ooag021_Supplementary_Data [file ooag021_supplementary_data.zip › Supplemental Figure Dashboard.docx]

**Supplemental Figure**

**Title:**
Sugammadex Merck Trial Dashboard Overview


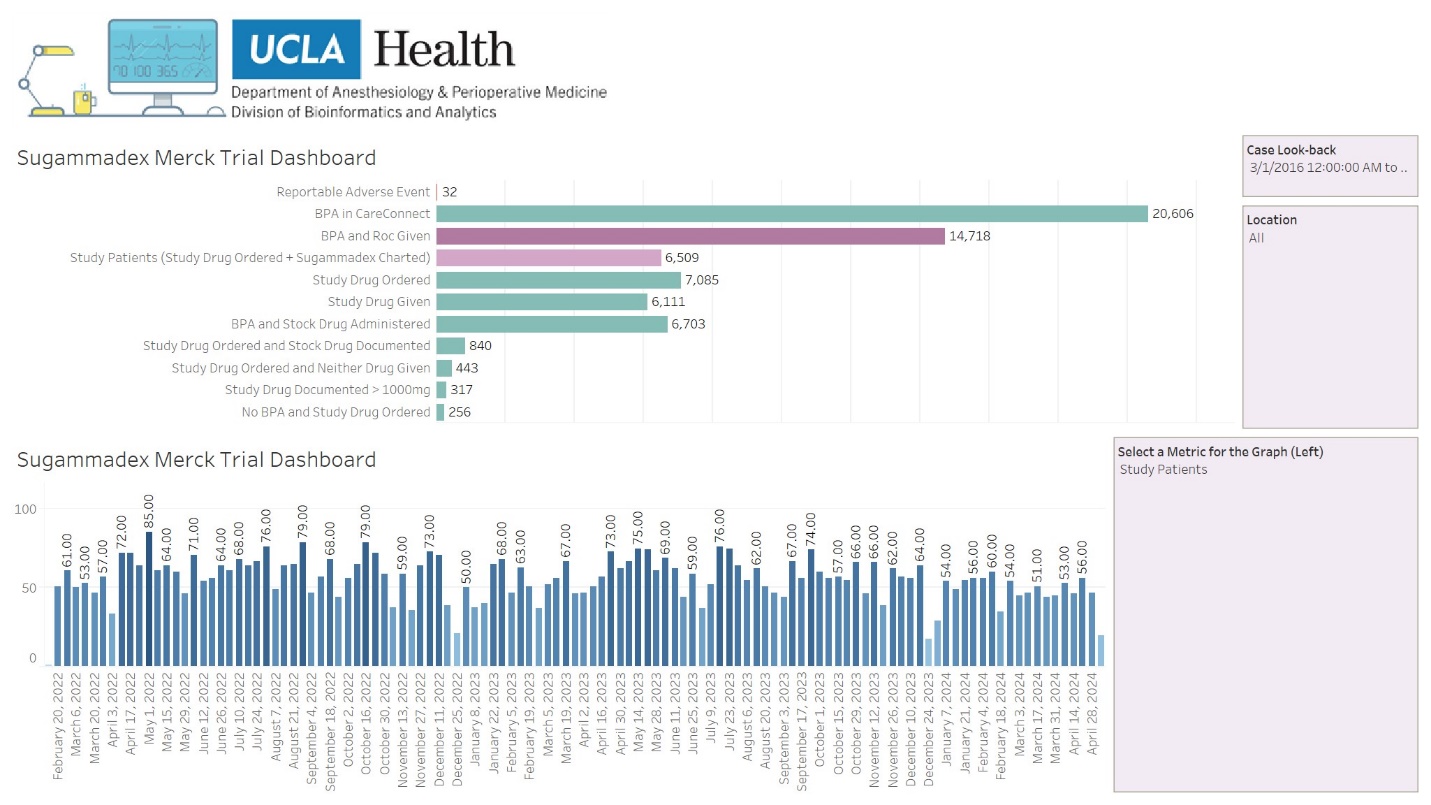
**Caption:**
This figure presents a sample view of the real-time dashboard used to monitor system performance throughout the study. The upper bar chart displays key operational metrics, including the number of Best Practice Advisory (BPA) alerts generated in the Epic electronic health record (EHR; CareConnect), study-drug orders placed through the automated workflow pathway, and documented drug administrations. The dashboard also highlights patterns such as frequent alerts among providers with lower rates of rocuronium administration, as well as variation in overall provider adherence. Built using data from the Perioperative Data Warehouse and visualized through Tableau Server, the dashboard enabled continuous oversight of system activity, workflow trends, and data quality on a weekly basis.
