# Supplementary material for: Automating eligibility assessment and enrollment for sugammadex administration within an integrated perioperative workflow
Source: JAMIA Open. 2026 Feb 17;9(1):ooag021. doi: 10.1093/jamiaopen/ooag021 (PMC12932941; doi:10.1093/jamiaopen/ooag021)
Supplement: ooag021_Supplementary_Data [file ooag021_supplementary_data.zip › Supplementary Table 1 Group Comp.docx]

**Supplementary Table 1**

**Title:** Comparison of Demographic, Clinical Characteristics, and Outcomes Between Post-Trial and Patients Missed to Non-Compliance

**Caption:** This table summarizes demographic characteristics, clinical comorbidities, and perioperative outcomes for patients whose providers adhered to the Best Practice Advisory (BPA)–linked study workflow (N = 5,424) compared with those whose providers bypassed the automated enrollment pathway (N = 5,168). Variables include sex, age, race/ethnicity, body mass index (BMI), weight, American Society of Anesthesiologists (ASA) physical status classification, and relevant clinical indicators such as preoperative oxygen saturation below 95% (SpO₂ <95%), obstructive sleep apnea (OSA), recent acute respiratory infection, and documented airway pathology. Perioperative outcomes include unexpected intensive care unit (ICU) transfers, reintubation, post-anesthesia care unit (PACU) length of stay, and minimum PACU oxygen saturation. Overall, demographic, and clinical characteristics were similar between groups, suggesting minimal evidence of systematic patient-selection differences associated with provider adherence behavior.

| Post vs Missed Patients |  | POST-TRIAL  (N=5424) | POST-TRIAL MISSED  (N=5168) | P-value |
| --- | --- | --- | --- | --- |
| Sex |  |  |  | 0.357 |
|  | F | 2844 (52.4%) | 2720 (52.6%) |  |
|  | M | 2576 (47.5%) | 2448 (47.4%) |  |
|  | Other | 4 (0.1%) | 0 |  |
| Age |  |  |  | <0.001 |
|  | Mean (SD) | 57.4 (17.0) | 58.6 (17.4) |  |
|  | Median (Q1, Q3) | 60.0 (45.0, 70.0) | 61.0 (46.0, 72.0) |  |
| Race |  |  |  | 0.587 |
|  | AFRICAN BLACK | 400 (7.4%) | 353 (6.8%) |  |
|  | ASIAN | 508 (9.4%) | 463 (9.0%) |  |
|  | WHITE | 3119 (57.5%) | 2989 (57.8%) |  |
|  | MIDDLE EASTERN NORTH AFRICAN | 195 (3.6%) | 180 (3.5%) |  |
|  | NATIVE AMERICAN INDIGENOUS | 60 (1.1%) | 44 (0.9%) |  |
|  | PACIFIC ISLANDER | 28 (0.5%) | 27 (0.5%) |  |
|  | UNKNOWN DECLINED TO SPECIFY | 1114 (20.5%) | 1112 (21.5%) |  |
| ASA Score |  |  |  | 0.056 |
|  | 1 | 84 (1.5%) | 75 (1.5%) |  |
|  | 2 | 1314 (24.2%) | 1370 (26.5%) |  |
|  | 3 | 3605 (66.5%) | 3342 (64.7%) |  |
|  | 4 | 421 (7.8%) | 380 (7.4%) |  |
| ASA Emergent Status |  |  |  | 0.005 |
|  | No | 5328 (98.2%) | 5035 (97.4%) |  |
|  | Yes | 96 (1.8%) | 133 (2.6%) |  |
| BMI |  |  |  | <0.001 |
|  | Mean (SD) | 30.4 (9.4) | 29.6 (9.4) |  |
|  | Median (Q1, Q3) | 28.3 (24.1, 35.5) | 27.9 (23.8, 33.8) |  |
| Weight in Kg |  |  |  | <0.001 |
|  | Mean (SD) | 87.0 (26.1) | 84.2 (24.5) |  |
|  | Median (Q1, Q3) | 83.0 (68.0, 102.0) | 81.0 (67.0, 98.0) |  |
| Anesthesia Duration |  |  |  | <0.001 |
|  | Mean (SD) | 228.9 (133.0) | 212.3 (125.7) |  |
|  | Median (Q1, Q3) | 196.0 (134.0, 287.0) | 180.0 (126.0, 262.0) |  |
| Obstruction Lung Disease |  |  |  | 0.034 |
|  | No | 3131 (57.7%) | 2877 (55.7%) |  |
|  | Yes | 2293 (42.3%) | 2291 (44.3%) |  |
| OSA |  |  |  | 0.897 |
|  | No | 3856 (71.1%) | 3680 (71.2%) |  |
|  | Yes | 1568 (28.9%) | 1488 (28.8%) |  |
| Preoperative SpO2 Less Than 95% |  |  |  | 0.067 |
|  | No | 4733 (87.3%) | 4447 (86.0%) |  |
|  | Yes | 691 (12.7%) | 721 (14.0%) |  |
| Acute Respiratory Infection |  |  |  | 0.032 |
|  | No | 4960 (91.4%) | 4663 (90.2%) |  |
|  | Yes | 464 (8.6%) | 505 (9.8%) |  |
| Airway Pathology |  |  |  | 0.576 |
|  | No | 4972 (91.7%) | 4721 (91.4%) |  |
|  | Yes | 452 (8.3%) | 447 (8.6%) |  |
| Historical Multiple Intubation Attempts |  |  |  | 0.033 |
|  | No | 4574 (84.3%) | 4279 (82.8%) |  |
|  | Yes | 850 (15.7%) | 889 (17.2%) |  |
| Abdominal, ENT, or Thoracic Surgery |  |  |  | 0.136 |
|  | No | 3707 (68.3%) | 3602 (69.7%) |  |
|  | Yes | 1717 (31.7%) | 1566 (30.3%) |  |
| Hemoglobin <10 in the Previous 6 Months |  |  |  | 0.547 |
|  | No | 4727 (87.1%) | 4483 (86.7%) |  |
|  | Yes | 697 (12.9%) | 685 (13.3%) |  |
| Upgrade from Floor to ICU Bed |  |  |  | 0.685 |
|  | No | 5372 (99.0%) | 5123 (99.1%) |  |
|  | Yes | 52 (1.0%) | 45 (0.9%) |  |
| Reintubation |  |  |  | 1 |
|  | No | 5398 (99.5%) | 5144 (99.5%) |  |
|  | Yes | 26 (0.5%) | 24 (0.5%) |  |
| Death |  |  |  | <0.001 |
|  | No | 5411 (99.8%) | 5168 (100.0%) |  |
|  | Yes | 13 (0.2%) | 0 |  |
| Recovery PACU Minutes |  |  |  | 0.805 |
|  | Mean (SD) | 145.3 (85.7) | 144.9 (85.5) |  |
|  | Median (Q1, Q3) | 124.0 (87.0, 181.0) | 124.0 (86.0, 181.0) |  |
| PACU Minimum SpO2 Value |  |  |  | 0.869 |
|  | Mean (SD) | 93.6 (2.8) | 93.6 (3.0) |  |
|  | Median (Q1, Q3) | 94.0 (92.0, 95.0) | 94.0 (92.0, 95.0) |  |
