# Supplementary material for: Automating eligibility assessment and enrollment for sugammadex administration within an integrated perioperative workflow
Source: JAMIA Open. 2026 Feb 17;9(1):ooag021. doi: 10.1093/jamiaopen/ooag021 (PMC12932941; doi:10.1093/jamiaopen/ooag021)
Supplement: ooag021_Supplementary_Data [file ooag021_supplementary_data.zip › Supplementary Table 2 Sugammadex Outcomes.docx]

**Supplementary Table 2**

**Title:**
Multivariable Analysis of Perioperative Outcomes Among Patients Meeting Automated Eligibility Criteria

**Caption:** This table presents multivariable analyses of four perioperative outcomes—unplanned transfers from the surgical floor to the intensive care unit (ICU), reintubation, post-anesthesia care unit (PACU) length of stay, and minimum PACU oxygen saturation—among patients who met the automated respiratory-risk eligibility criteria. The models evaluate associations between these outcomes and relevant demographic, clinical, and procedural variables, including sex, age, American Society of Anesthesiologists (ASA) physical status classification, body mass index (BMI), obstructive sleep apnea (OSA), and preoperative oxygen saturation below 95% (SpO₂ <95%). Odds ratios (ORs), effect estimates, and 95% confidence intervals (CIs) are reported, with statistically significant p-values (p < 0.05) indicated. These analyses are intended to contextualize perioperative outcomes within the eligible patient population and are not designed to compare medication effects or assess treatment efficacy.

|  | FLOOR TO ICU UPGRADE | REINTUBATION | PACU RECOVERY MINUTES | PACU MINIMUM SPO2 |
| --- | --- | --- | --- | --- |
| Overall | 0.65 (0.45 - 0.93),  0.019 | 0.58 (0.34 - 0.97), 0.037 | -16.3 (-19.6, -13.1), <0.001 | -0.64 (-0.76, -0.51), <0.001 |
| Male | 0.84 (0.56 - 1.26),  0.389 | 0.45 (0.26 - 0.78),  0.005 | -11.3 (-14.6, -8.08),  <0.001 | 0.05 (-0.08, 0.17),  0.449 |
| Age | 1.02 (1.01 - 1.03),  0.002 | 1.02 (1.00 - 1.03),  0.025 | 0.14 (0.04, 0.24),  0.007 | -0.02 (-0.03, -0.02),  <0.001 |
| ASA Score | 1.59 (1.16 - 2.17),  0.004 | 2.23 (1.41 - 3.53),  0.001 | -3.14 (-6.02, -0.26),  0.033 | -0.17 (-0.28, -0.06),  0.003 |
| ASA Emergent Status | 3.43 (1.90 - 6.18),  <0.001 | 2.39 (1.02 - 5.60),  0.044 | -37.40 (-47.9, -26.8),  <0.001 | 0.10 (-0.30, 0.50),  0.621 |
| BMI | 0.92 (0.86 - 0.97),  0.005 | 0.85 (0.79 - 0.93),  <0.001 | 0.00 (-0.01, 0.01),  0.769 | 0.00 (0.00, 0.00),  0.595 |
| Weight in Kg | 1.02 (1.00 - 1.04),  0.045 | 1.04 (1.02 - 1.06),  0.001 | 0.06 (-0.01, 0.13),  0.104 | -0.02 (-0.02, -0.02),  <0.001 |
| Anesthesia Duration | 1. (1.00 - 1.00),   <0.001 | 1.00 (1.00 - 1.00),  <0.001 | -0.02 (-0.03, -0.01),  0.004 | 0.00 (0.00, 0.00),  0.008 |
| Obstruction Lung Disease | 1.01 (0.72 - 1.43),  0.938 | 1.84 (1.16 - 2.95),  0.010 | 4.16 (0.83, 7.49),  0.014 | -0.23 (-0.36, -0.11),  <0.001 |
| OSA | 0.54 (0.32 - 0.91),  0.02 | 0.41 (0.18 - 0.93),  0.032 | 7.10 (3.24, 10.9),  <0.001 | -0.18 (-0.32, -0.03),  0.018 |
| Preoperative SpO2 Less Than 95% | 2.30 (1.53 - 3.45),  <0.001 | 1.83 (1.00 - 3.35),  0.052 | 3.08 (-2.12, 8.28),  0.245 | -1.42 (-1.62, -1.22),  <0.001 |
| Acute Respiratory Infection | 0.76 (0.42 - 1.37),  0.362 | 0.75 (0.34 - 1.62),  0.458 | -3.49 (-8.87, 1.88),  0.203 | -0.03 (-0.23, 0.17), 0.77 |
| Airway Pathology | 5.90 (4.19 - 8.31),  <0.001 | 31.6 (17.5 - 57.1),  <0.001 | -0.53 (-5.67, 4.61),  0.840 | 0.13 (-0.06, 0.33),  0.180 |
| Historical Multiple Intubation Attempts | 1.23 (0.85 - 1.78),  0.269 | 0.62 (0.34 - 1.14),  0.122 | -2.51 (-6.26, 1.25),  0.191 | 0.11 (-0.03, 0.25),  0.137 |
| Abdominal, ENT, or Thoracic Surgery | 0.48 (0.31 - 0.74),  0.001 | 0.29 (0.15 - 0.56),  <0.001 | -10.95 (-14.42, -7.48),  <0.001 | -0.10 (-0.24, 0.03),  0.124 |
| Hemoglobin <10 in the Previous 6 Months | 2.16 (1.52 - 3.08),  <0.001 | 1.46 (0.89 - 2.38),  0.132 | -13.13 (-17.87, -8.39),  <0.001 | 0.80 (0.62, 0.98),  <0.001 |
